# scGO: interpretable deep neural network for cell status annotation and disease diagnosis

**DOI:** 10.1093/bib/bbaf018

**Published:** 2025-01-16

**Authors:** You Wu, Pengfei Xu, Liyuan Wang, Shuai Liu, Yingnan Hou, Hui Lu, Peng Hu, Xiaofei Li, Xiang Yu

**Affiliations:** School of Life Sciences and Biotechnology, Shanghai Jiao Tong University, No. 800 Dong Chuan Road, Shanghai 200240, China; School of Life Sciences and Biotechnology, Shanghai Jiao Tong University, No. 800 Dong Chuan Road, Shanghai 200240, China; School of Agriculture and Biology, Shanghai Jiao Tong University, No. 800 Dong Chuan Road, Shanghai 200240, China; School of Life Sciences and Biotechnology, Shanghai Jiao Tong University, No. 800 Dong Chuan Road, Shanghai 200240, China; School of Agriculture and Biology, Shanghai Jiao Tong University, No. 800 Dong Chuan Road, Shanghai 200240, China; School of Life Sciences and Biotechnology, Shanghai Jiao Tong University, No. 800 Dong Chuan Road, Shanghai 200240, China; Ministry of Education, Shanghai Ocean University, No. 999, Huchenghuan Road, Shanghai 201306, China; School of Life Sciences and Biotechnology, Shanghai Jiao Tong University, No. 800 Dong Chuan Road, Shanghai 200240, China; Shanghai Pudong New Area People’s Hospital, No. 490, Chuanhuan South Road, Shanghai 201299, China; School of Life Sciences and Biotechnology, Shanghai Jiao Tong University, No. 800 Dong Chuan Road, Shanghai 200240, China

**Keywords:** scRNA-seq, sparse neural networks, interpretable model, cell status annotation, disease diagnosis

## Abstract

Machine learning has emerged as a transformative tool for elucidating cellular heterogeneity in single-cell RNA sequencing. However, a significant challenge lies in the “black box” nature of deep learning models, which obscures the decision-making process and limits interpretability in cell status annotation. In this study, we introduced scGO, a Gene Ontology (GO)–inspired deep learning framework designed to provide interpretable cell status annotation for scRNA-seq data. scGO employs sparse neural networks to leverage the intrinsic biological relationships among genes, transcription factors, and GO terms, significantly augmenting interpretability and reducing computational cost. scGO outperforms state-of-the-art methods in the precise characterization of cell subtypes across diverse datasets. Our extensive experimentation across a spectrum of scRNA-seq datasets underscored the remarkable efficacy of scGO in disease diagnosis, prediction of developmental stages, and evaluation of disease severity and cellular senescence status. Furthermore, we incorporated *in silico* individual gene manipulations into the scGO model, introducing an additional layer for discovering therapeutic targets. Our results provide an interpretable model for accurately annotating cell status, capturing latent biological knowledge, and informing clinical practice.

## Introduction

Single-cell RNA-sequencing (scRNA-seq) has emerged as a powerful technology for studying cellular heterogeneity [1–9]. Cell annotation represents a pivotal and complicated step in scRNA-seq analysis. Several methodologies have been proposed to address this challenge, including marker gene-based, similarity-based, and machine learning–based approaches [10–20]. However, each of these methods possesses inherent limitations.

Marker gene–based methods for scRNA-seq cell type annotation rely on the expression of specific genes uniquely associated with particular cell types [14, 21, 22]. Similarity-based methods, such as singleR [11], annotate cell types by measuring the correlation of gene expression patterns between the query cells and a reference dataset. The accuracy of the annotation is notably influenced by the quality and representativeness of the reference dataset [23, 24]. The increasing availability of large-scale scRNA-seq datasets has led to significant advancements in cell type annotation using deep learning approaches [25–32]. However, deep learning models operate as black boxes, processing input data to generate predictions without revealing the biological mechanisms that define specific cell types [33–35]. This lack of transparency limits the ability to interpret and understand the rationale behind the predictions [36–38].

Knowledge-based neural networks, such as P-net [39], KPNN [40], expiMAP [41], dCell [42], GOWDL [43], and Elektrum [44], have demonstrated their capability to enhance interpretability and provide biologically meaningful insights across various domains of biological and medical research. The Gene Ontology (GO) Knowledgebase is a comprehensive repository that serves as the world’s largest source of information on the functions of genes, providing structured and computable information regarding the functions and products of genes [45, 46]. Transcription factors (TFs) are key regulators of gene expression, and DNA affinity purification sequencing (DAP-seq) has revealed genome-wide TF-targeting genes [47]. However, the knowledge combining GO and TF-binding networks for interpretable cell annotation has not been well characterized. In this study, we aim to bridge this gap by leveraging these powerful resources to create a more comprehensive, interpretable framework for studying cellular heterogeneity and annotating cell types with enhanced precision.

To achieve this, we propose scGO, a GO-inspired model that integrates GO data with TF-binding potential. The primary advantage of scGO lies in its ability to combine biological knowledge with deep learning, achieving both high accuracy and enhanced interpretability. Additionally, scGO adopts sparse neural network architecture, significantly reducing the number of parameters required. This design not only improves computational efficiency but also mitigates the risk of overfitting, enabling the model to generalize effectively across diverse datasets. However, scGO also has potential limitations. Its reliance on high-quality GO and TF-binding data may pose challenges, particularly for species with less well-characterized or incomplete annotations of GO terms and TF-binding profiles.

In this study, we develop a sparse and interpretable deep learning model, scGO, integrating GO and TF-binding potential, which shows less computational cost compared to a full-dense model. We then systematically evaluate its performance across various datasets, showing that scGO outperforms existing state-of-the-art methods. Furthermore, we demonstrate that scGO provides interpretable prediction results, identifying the top-ranked genes, GO terms, and TF-binding events that contribute most to the model’s predictions. Finally, we apply scGO to a wide range of biological tasks and species, including disease-level diagnosis, therapeutic target discovery, developmental stage prediction, and cell senescence evaluation.

## Methods

### The construction of scGO model

**Figure 1:**
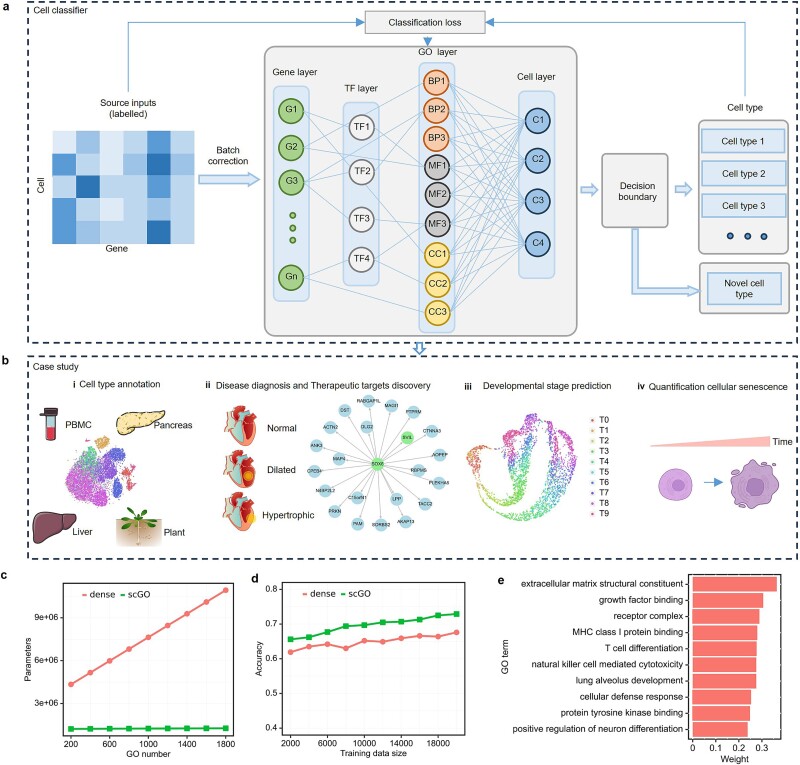
Overview of scGO model. (a) The architecture of scGO model. (b) The application of scGO in this research, including cell type annotation, cell disease diagnosis, therapeutic target discovery, developmental stage prediction, and cellular senescence quantification. (c) Comparison of scGO with dense-connected neural networks. The parameters in scGO are significantly reduced compared to those in dense-connected neural networks. (d) The accuracy of scGO surpasses that of dense networks, given the training size ranging from 2 k to 20 k. (e) The contribution of specific GO terms to a cell type can be indicated by tracing the associated weights.

The scGO model employs a sparse connection structure within its neural network, where each node corresponds to a specific biological entity, such as a gene, a TF, a GO term, or a cell type (Supplementary Fig. 1 and Fig. 1a). Beyond its primary application in cell type annotation, scGO can also be applied to a variety of tasks, such as disease diagnosis and developmental stage prediction (Fig. 1b).

Within the GO layer, 1946 nodes encapsulate functional annotations across molecular functions, biological processes, and cellular components. TFs in the TF layer serve as intermediaries, connecting the gene input layer with the GO layer. These TF nodes transduce the expression patterns of regulatory genes into the ontology layer, reflecting their regulatory roles. At the top of the architecture is the cell type layer, which consists of distinct nodes representing a range of cell types, marking the culmination of scGO’s computational process.

During the training process, the weights of the connections are dynamically adjusted to optimize the model’s performance. This adjustment activates the most relevant connections that contribute to the final output. By tracing these activated connections and analyzing their weights, the model identifies specific input elements, such as genes or pathways, that significantly influence the results. This enhances scGO’s interpretability and ensures that its predictions are grounded in underlying biological principles. In training the scGO model, an Adam optimizer with a fixed learning rate of 0.001 was employed. Cross-entropy was used as the classification loss:


$$ Loss=-\sum_{i=1}^n{z}_i\log \left({q}_i\right) $$

where ${z}_i$ is the ground-truth cell type label of cell $i$. ${q}_i$ is the output node value of cell $i$.

The performance of scGO was evaluated by the accuracy, F1-score, and area under the receiver-operating characteristic (ROC) curve. Accuracy measures the proportion of correct predictions made by the model compared to the total number of predictions.


$$ \text{Accuracy}=\frac{\text{TP}+\text{TN}}{\text{TP}+\text{TN}+\text{FP}+\text{FN}} $$

where:

TP = true positives (correctly predicted positive class).

TN = true negatives (correctly predicted negative class).

FP = false positives (incorrectly predicted as positive).

FN = false negatives (incorrectly predicted as negative).

In real-world scRNA-seq data, the class distribution is often imbalanced, which makes the F1-score a useful metric for evaluating model performance. The F1-score is the harmonic mean of precision and recall and is calculated as follows:


$$ \text{F}1-\text{score}=2\cdot \frac{\text{Precision}\cdot \text{Recall}}{\text{Precision}+\text{Recall}} $$

where:


$$ \text{Precision}=\frac{\text{TP}}{\text{TP}+\text{FP}} $$

$$ \text{Recall}=\frac{\text{TP}}{\text{TP}+\text{FN}} $$

The ROC curve was created by plotting the true positive rate (TPR) and the false positive rate (FPR) at various threshold settings.


$$ \text{True}\ \text{positive}\ \text{rate}=\frac{\text{TP}}{\text{TP}+\text{FN}} $$

$$ \text{False}\ \text{positive}\ \text{rate}=\frac{\text{FP}}{\text{FP}+\text{TN}} $$

A higher area under the curve (AUC) indicates better performance as it means the model is more capable of distinguishing between positive and negative classes.

### Gene Ontology knowledge utilization

In scGO, GO knowledge is employed to establish connections between genes and GO nodes. A gene node is connected to a GO node if the gene is annotated with the GO term. To achieve sparsity in connections, we introduced a binary mask matrix, denoted as ${M}^{GO}$, into the linear layer connecting the gene and GO layers. Each element ${M}_{ij}^{GO}$ in the matrix is set to 1 if gene *i* is annotated with GO term *j* and 0 otherwise. This approach effectively prunes specific weight connections.

The human and Arabidopsis GO annotations used in this study are downloaded from the GO knowledgebase (https://doi.org/10.5281/zenodo.7504797) [46], which supports a total of 39 species.

### Transcription factor–binding knowledge utilization

The gene regulation knowledge employed in this study was derived from DNA-binding experiments involving transcriptional regulators. Specifically, human and *Arabidopsis thaliana* DAP-seq peaks data were retrieved from the Remap database [48]. Subsequently, we utilized the Hypergeometric Optimization of Motif EnRichment (HOMER) software (v4.11) [49] to annotate the peak data, selecting those annotated as transcription start site (TSS) for subsequent analysis.

### Batch correction and data integration

In scRNA-seq, batch effects can significantly impact the interpretation of scRNA-seq data and may lead to incorrect conclusions if not properly addressed. In this study, data from various sources was harmonized using the Seurat data integration pipeline [50]. Initially, log normalization was applied, and variable features were identified independently for each dataset. Subsequently, the “anchors,” representing matching cell populations across the individual datasets, were determined to combine these datasets into a unified Seurat object. This integration process aligns and harmonizes the data, allowing for coherent analysis across diverse sources.

### Evaluation of gene importance

We used a gradient-based method, DeepLIFT [51], to evaluate the feature importance in the gene layer. In Brief, DeepLIFT is a technique designed to dissect a neural network’s output prediction for a given input. It achieves this by tracing the influence of all neurons in the network on each input feature. DeepLIFT accomplishes this by comparing each neuron’s activation to a “reference activation” and then assigning contribution scores based on the disparity between these values.

### *In silico* gene perturbation

The assessment involves performing *in silico* gene perturbation to evaluate the consequences of inhibiting or activating a particular gene within the scGO model on the anticipated cell status. In this investigation’s context of the cardiomyopathy disease scGO model, *in silico* perturbation involves manipulating single gene expression of normal myocardial cells. In the case of *in silico* inhibition, gene expression is scaled by 0.5; in the case of *in silico* gene activation, gene expression is scaled by 2.

## Results

### Biological knowledge inspired sparse connections within the scGO model

Previous studies have shown that sparse and interpretable neural networks could perform better than fully connected models [41, 52–54]. In contrast to fully connected neural networks, the connectivity in scGO is specifically tailored based on GO terms and TF regulatory knowledge, which significantly reduces the number of parameters (Fig. 1c). This sparsity not only requires less training data but also mitigates the risk of overfitting (Supplementary Fig. 2e and f). As a result, scGO can achieve accurate cell type annotation with smaller training data size, leading to improved generalization performance (Fig. 1d). We conducted a systematic analysis of memory usage, running time, and accuracy across varying numbers of genes and GO terms using the Baron dataset [55] (Supplementary Fig. 2a–d).

In addition to its enhanced performance, scGO provides the unique capability to elucidate the specific biological entities or processes that contribute to its outcomes by inverse tracing of weights (Fig. 1e). Except for the known TF–target pairs, we incorporate a *de novo* TF–target mode into scGO to infer TF regulations (Supplementary Fig. 2). We adopt a strategy in which the TF expression is embedded into a latent space, which is subsequently projected onto the target RNA expression space. Following this, we performed *in silico* perturbations for each TF and inferred the resulting RNA expression fluctuations to predict TF–target regulatory interactions. This approach provides a comprehensive biological context, enabling us to unravel the intricate connections between gene expressions, regulatory networks, and functional annotations, ultimately enriching our understanding of the underlying biological mechanisms.

### High performance of scGO across datasets and tissues

**Figure 2:**
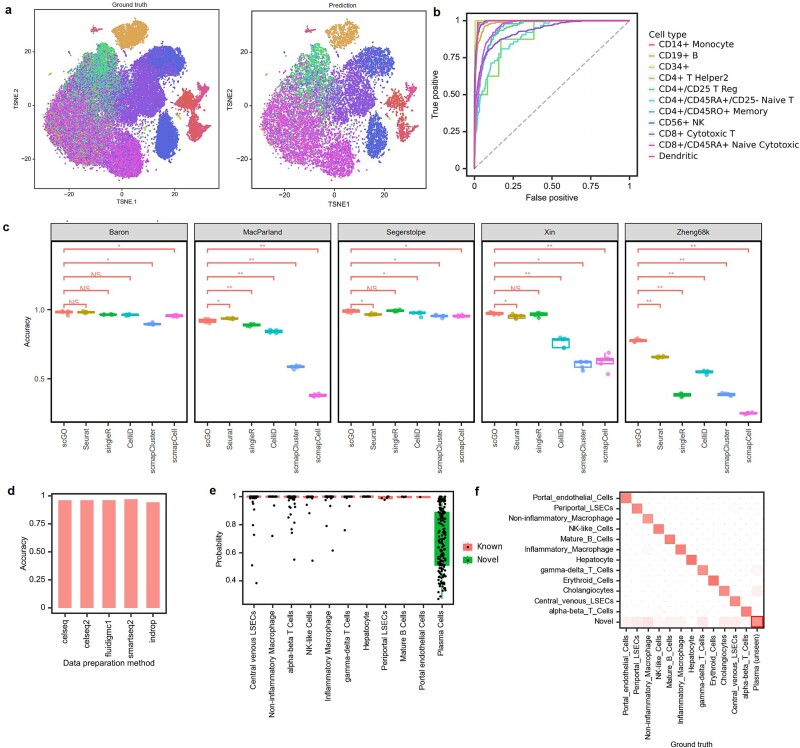
Performance of scGO across datasets compared to existing tools. (a) Visualization of the Zheng68K dataset using t-Distributed Stochastic Neighbor Embedding (t-SNE). Left panel is colored based on marker-based classifications from the original research. Right panel is colored according to scGO predicted cell types. (b) ROC curves for each cell type prediction of the scGO model. (c) Performance comparison of scGO with Seurat, singleR, CelliD, scmapCluster, and scmapCell on five datasets (Zheng68K, Baroon, Segerstolpe, MacParland, and Xin). All statistical analyses use two-sided Wilcoxon tests. The significance levels are as follows: * represents *P* < .05, ** represents *P* < .01, and *** represents *P* < .001. (d) Performance evaluation of scGO across datasets generated from five different experimental protocols. (e) Confidence probabilities distribution for scGO predictions. (f) Heatmap demonstrating the successful classification of plasma cells as a novel cell type by scGO.

To evaluate the performance of scGO, we first tested scGO on a human peripheral blood mononuclear cells Zheng68K dataset [56] containing 68 589 cells of 11 immune cell populations (Fig. 2a). scGO achieved an overall accuracy of 0.77, demonstrating robust performance (Fig. 2b). Compared to to Seurat [50] and CelliD [57], two widely used RNA analysis tools, scGO showed superior performance in identifying CD4+/CD25 T Reg cell, CD8+ cytotoxic T cell, and dendritic cells. These cell types exhibit high similarity and have proven challenging to distinguish in prior studies [56].

We further compared the performance of scGO with five state-of-the-art cell type annotation methods (Seurat [50], singleR [11], celliD [57], scmapcluster [58], and scmapcell [58]) on five datasets (Zheng68k [56], Baron [55], Segerstolpe [59], MacParland [60], and Xin [61]). We employed a 5-fold cross-validation strategy for each dataset to evaluate the performance and robustness of these methods. On average, scGO consistently ranked as the top-performing method among the six cell annotation methods in terms of accuracy and F1-score across all datasets (Fig. 2c and Supplementary Fig. 4).

To assess the generalization capability of scGO, we tested whether a pretrained scGO model could be effectively applied to single-cell data generated from different labs and platforms. Specifically, we selected human pancreas datasets generated through five single-cell preparation methods (celseq [62], celseq2 [62], fluidigmc1 [63], smartseq2 [59], and indrops [55]). To mitigate batch effects, the datasets generated from these five library preparation technologies were integrated using Seurat (Supplementary Fig. 5a and b). For cross-platform validation, we employed an iterative approach in which one of the five single-cell preparation methods was reserved as the test set, while the remaining four were used to train the scGO model. This approach ensured that each platform was independently evaluated in an out-of-sample manner. The results consistently exhibit high accuracy (ranging from 0.942 to 0.970) across all five unseen datasets, highlighting scGO’s ability to generalize effectively across diverse scRNA-seq data (Fig. 2d and Supplementary Fig. 5c–f).

We further examined the probability distribution of cell type predictions to assess scGO’s ability to identify novel cell types. We initially trained scGO on the MacParland dataset, excluding plasma cells. Subsequently, we evaluated the scGO model using both known cell types (new cells from the test set whose cell types were utilized during training) and a novel cell type (plasma cells). As expected, the prediction probabilities for known cell types were consistently high, whereas those for novel cell types remained low (Fig. 2e and Supplementary Fig. 6a and b). By applying a predefined cutoff threshold (0.95 in this case), scGO successfully classified 90.5% of plasma cells as a novel cell type (Fig. 2f).

### scGO provides interpretable prediction results

**Figure 3:**
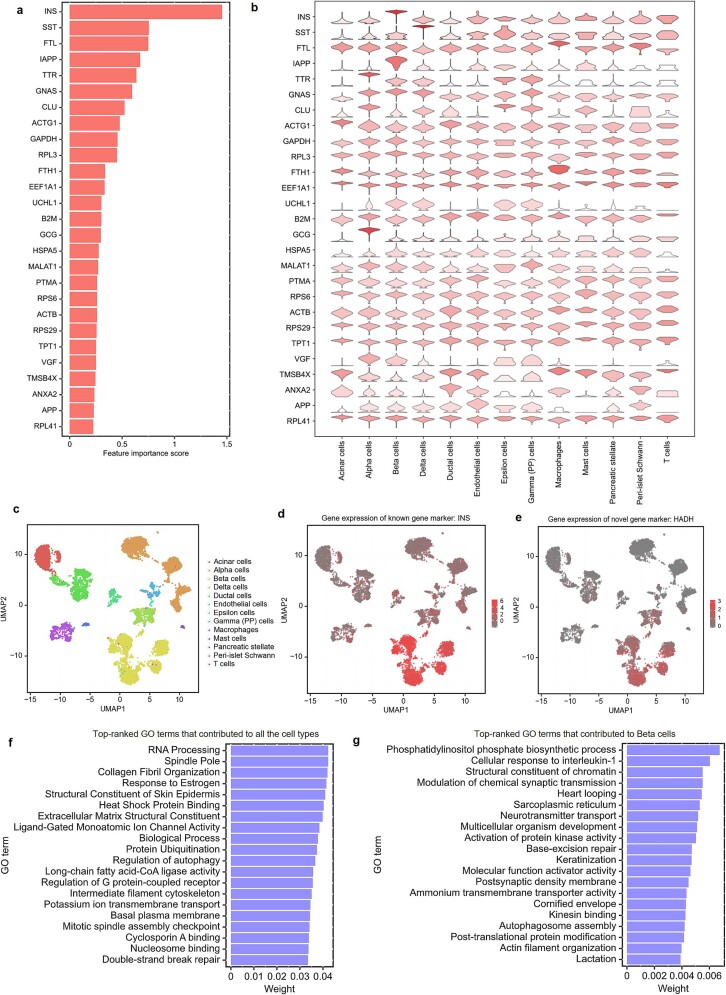
The interpretability of the scGO model. (a) Top-ranked important genes of scGO by DeepLIFT gradient method. The scGO model was trained on the Baron pancreas dataset. (b) The gene expression profiles of the top-ranked genes in each respective cell type. Among these genes, a substantial proportion are cell type–specific and can function as marker genes. This panel was generated by SCANPY [64]. (c) Uniform Manifold Approximation and Projection (UMAP) visualization of Baron dataset, which comprises 13 cell types. (d, e) Umap visualization showing the RNA abundance of *INS* (d) and *HADH* (e), which are highly expressed in beta cells [65]. (f, g) Top-ranked GO terms that contributed to all the cell types (f) and beta cells (g).

To enhance the interpretability of the model’s predictions, we employed a gradient-based method, DeepLIFT [51], to evaluate feature importance in the gene layer. Using the Baron pancreas dataset as an example, we first trained the scGO model to annotate 13 cell types. Then, we used DeepLIFT to decompose the output predictions by backpropagating the contributions of all neurons in the network to each feature in the gene layer, obtaining an importance score for each input gene (Fig. 3a). A significant proportion of the top-ranked genes exhibited high expression in specific cell types, making them strong candidates as marker genes of those cell types (Fig. 3b). Additionally, our analysis identified the well-established marker gene *INS* for beta cells, as well as *HADH*, which may represent a potential novel marker for pancreatic beta cells (Fig. 3c–e). By tracing back the model weights, we further elucidated the contributions of each GO term to the prediction results (Fig. 3f). For pancreatic beta cells, scGO identified key GO terms, including phosphatidylinositol phosphate biosynthesis, cellular response to interleukin-1, and modulation of synaptic transmission, all of which are associated with insulin secretion, a core function of beta cells (Fig. 3g). These results underscore scGO’s ability to provide valuable insights underlying cell type classifications.

### Cell disease diagnosis and therapeutic targets discovery

**Figure 4:**
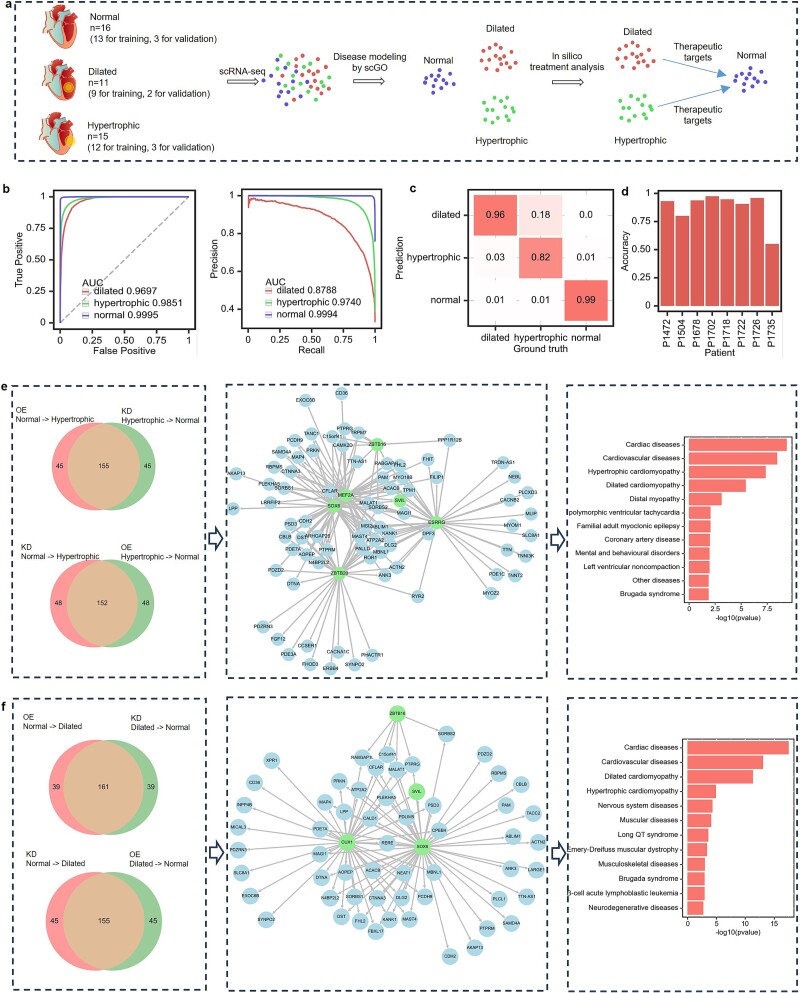
scGO for heart disease diagnosis and therapeutic targets discovery. (a) The scheme for heart disease diagnosis. The scGO model was trained on scRNA-seq data, combining normal cardiomyocytes with two types of diseased cells, to identify the disease states of heart muscle cells. Subsequently, the trained scGO disease model was employed for *in silico* gene perturbation to discover candidate therapeutic targets that could potentially shift the diseased cells toward a normal state. (b) The ROC curve and PR curve of the scGO disease model that identifies hypertrophic cells and dilated cells. (c) The confusion matrix displays the proportions between the actual and predicted disease states. (d) Barplot illustrating the accuracy of disease cell identification of the eight test donors by scGO. (e) Therapeutic target discovery for hypertrophic cardiomyocytes. Through *in silico* gene perturbation, scGO identified 155 genes whose inhibition shifts hypertrophic cells toward normal and 152 genes whose activation has a similar effect (left). Transcription factor regulatory network of disease risk genes contributing to hypertrophic cardiomyocytes (middle). Top-ranked GO terms affecting hypertrophic cardiomyocytes (right). (f) Therapeutic targets discovery for dilated cardiomyocytes. Through *in silico* gene perturbation, scGO identified 161 genes whose inhibition shifts dilated cells toward normal and 155 genes whose activation has a similar effect (left). Transcription factor regulatory network of disease risk genes contributing to dilated cardiomyocytes (middle). Top-ranked GO terms affecting dilated cardiomyocytes (right).

scRNA-seq holds significant promise for applications in disease diagnosis [66, 67]. Next, we explored the application of scGO in identifying diseased cells and discovering candidate therapeutic targets. Hypertrophic cardiomyopathy and dilated cardiomyopathy are prevalent and clinically significant heart diseases that pose substantial health challenges worldwide [68, 69]. In this case study, we applied scGO to diagnose heart failure states at the single-cell level and discover candidate therapeutic targets (Fig. 4a and Supplementary Fig. 7). First, we trained the scGO model on scRNA-seq data of heart diseases [70] to distinguish hypertrophic and dilated myocardial cells from normal myocardial cells (Supplementary Fig. 8). The scRNA-seq dataset used for this analysis comprised a total of 158 469 cardiac muscle cells originating from 42 individual donors. Among these donors, 16 individuals exhibited normal cardiac profiles, 15 presented hypertrophic conditions, and 11 had dilated conditions. We allocated cells from 8 donors (3 with normal cardiac profiles, 3 with hypertrophic conditions, and 2 with dilated conditions) as a hold set for independent validations. The overall out-of-sample accuracy was 92% (Fig. 4b–d).

Next, we performed *in silico* perturbation analysis [25], a computational approach that simulates gene alterations to identify genes associated with disease states and discover potential therapeutic targets. Specifically, we conducted *in silico* activation and inhibition of individual genes within the scGO model (Supplementary Fig. 9). In total, we identified 155 genes that, when deactivated, can induce a transition of hypertrophic cells toward a normal state, along with 152 genes whose activation achieves the same effect (Fig. 4e). Additionally, we found 161 genes that, when deactivated, facilitate a shift of dilated cells toward a normal state, accompanied by 155 genes whose activation accomplishes the same transition (Fig. 4f). Specifically, scGO pinpointed *MEF2A*, a recognized TF implicated in the development of cardiac hypertrophy [71, 72]. Moreover, *in silico* activation of *MEF2A* demonstrated the capability to redirect hypertrophic cells toward a normal state. Additionally, scGO identified genes such as *ZBTB20*, *ESRRG*, and *SOX6*, which have established links to the onset of cardiac hypertrophy [73–75]. scGO also identified *CUX1*, *SOX6*, and *SVIL*, which have been reported as pivotal factors implicated in causing dilated cardiomyopathy [76–78]. The identified disease-associated genes are enriched in cardiac diseases, hypertrophic cardiomyopathy, and dilated cardiomyopathy pathways (Fig. 4e and f).

**Figure 5:**
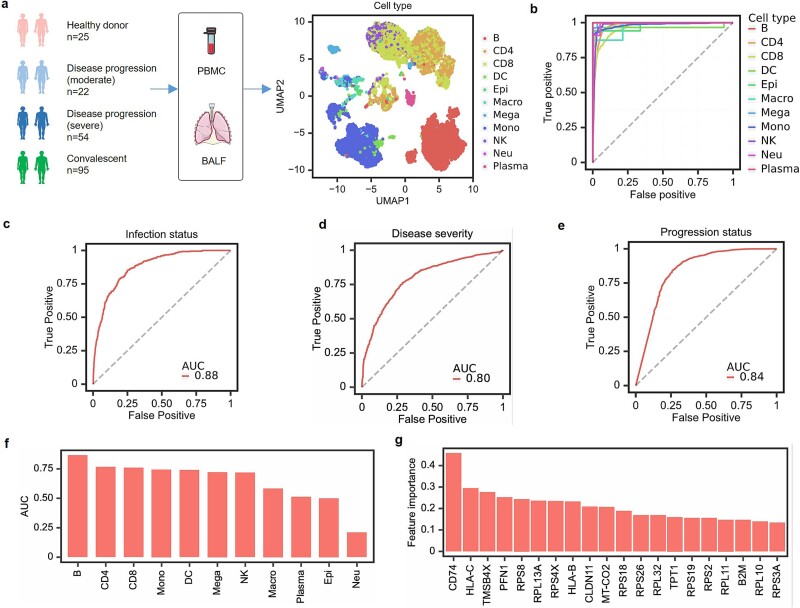
Evaluation of disease severity using scGO. (a) A schematic overview of the COVID-19 dataset used in this study. (b) ROC curves of scGO for cell type identification. (c–e) ROC curve of scGO for infection prediction (c), disease severity classification (d), and disease progression evaluation (e). (f) Evaluation of disease severity for each cell type. (g) Top 20 genes identified by scGO contributing to disease severity prediction.

We further assessed the ability of scGO to evaluate disease severity. This experiment used a COVID-19 scRNA-seq atlas [79] containing 184 samples collected from 39 institutes or hospitals (Fig. 5a). We employed an out-of-sample strategy to evaluate scGO’s performance on this COVID-19 dataset. Specifically, we used scRNAseq data from 146 donors as the training set and the remaining 50 donors as the test set. scGO identified 11 cell types with high accuracy in the test set (Fig. 5b). We then investigated whether scGO could distinguish infected cells from normal cells and further differentiate disease severity (severe or moderate), as well as identify whether the patient was in the progression or convalescence phase. scGO achieved ROC-AUC scores of 0.88, 0.80, and 0.84 across the three tasks, demonstrating its ability to assess disease severity (Fig. 5c–e). We also examined each cell type and found that B cells, CD4 T cells, CD8 T cells, and monocytes made the most significant contributions to disease severity evaluation (Fig. 5f). Furthermore, scGO identified the top 20 genes by feature importance, which were key contributors to the prediction results (Fig. 5G). These findings highlight scGO’s strong performance in disease diagnosis and severity assessment.

### scGO uncovers developmental stages in plants

**Figure 6:**
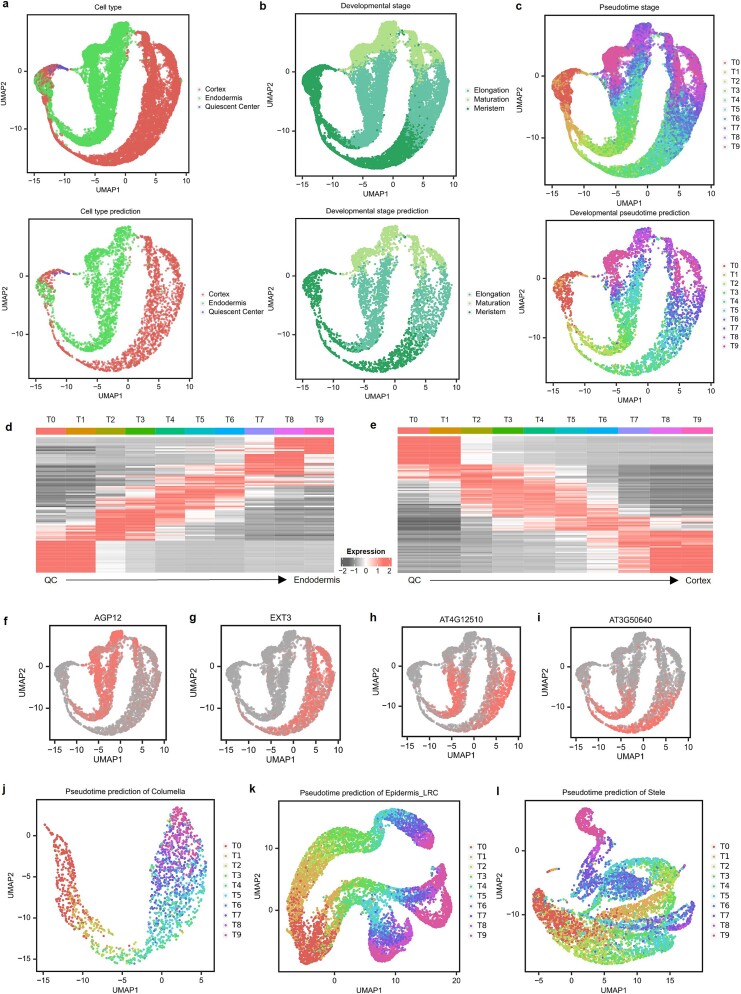
Application of scGO in a plant to predict developmental stage. (a–c) scGO accurately identified cell types (a), developmental stages (b), and accurately predicted consensus pseudotime (c) in Arabidopsis ground tissue. (d) Visualization of scaled expression profiles of differentially expressed genes in the endodermis (d) and the cortex (e) over predicted pseudotime. (f, g) Marker genes identified by scGO for cell type endodermis (f) and cortex (g). (h, i) Marker genes identified by scGO for developmental stage elongation (h) and meristem (i). (j–l) Application of scGO to identify pseudotime stage to columella (j), epidermis (k), and stele (l) tissues.

scGO demonstrated high performance in human cell lines, prompting us to explore its generalizability across species. We applied scGO to the Arabidopsis root atlas [80] to identify cell types and developmental stages. scGO successfully distinguished three ground tissue cell types—meristem, elongation, and maturation stages. The model achieved 0.972 accuracy for cell type identification (Fig. 6a) and 0.947 accuracy for developmental stage prediction (Fig. 6b). Using DeepLIFT, we identified key marker genes associated with each cell type and stage. Notable cortex-specific gene *EXT3* was identified as a marker for cortex cells (Fig. 6g), while *AGP12* marked endodermis cells (Fig. 6f). Furthermore, scGO revealed *AT4G12510* as a marker gene for the elongation stage and *AT3G50640* as a marker gene for the meristem stage (Fig. 6h and i).

Subsequently, we investigated whether scGO could delineate the developmental stage in the form of pseudotime, a computational representation that orders cells along a trajectory based on their gene expression profiles. The consensus pseudotime [80] labels were derived from averaging the results of both CytoTRACE [81] and scVelo [82], two tools widely used to model cellular trajectories and dynamics. These consensus pseudotime labels were subsequently partitioned into 10 groups, denoted as T0 to T9. The pseudotime status represented by scGO’s predictions aligned well with the actual pseudotime values. (Fig. 6c). The differential gene expression observed across the predicted pseudotime bins exhibited a gradual progression pattern (Fig. 6d and e), closely mirroring the original research findings [80]. We next applied scGO to stele, columella, and epidermal label-retaining cells to predict consensus pseudotime (Fig. 6j–l), and the results demonstrated consistency across these diverse tissues.

### Quantifying cellular senescence through the regression mode of scGO

**Figure 7:**
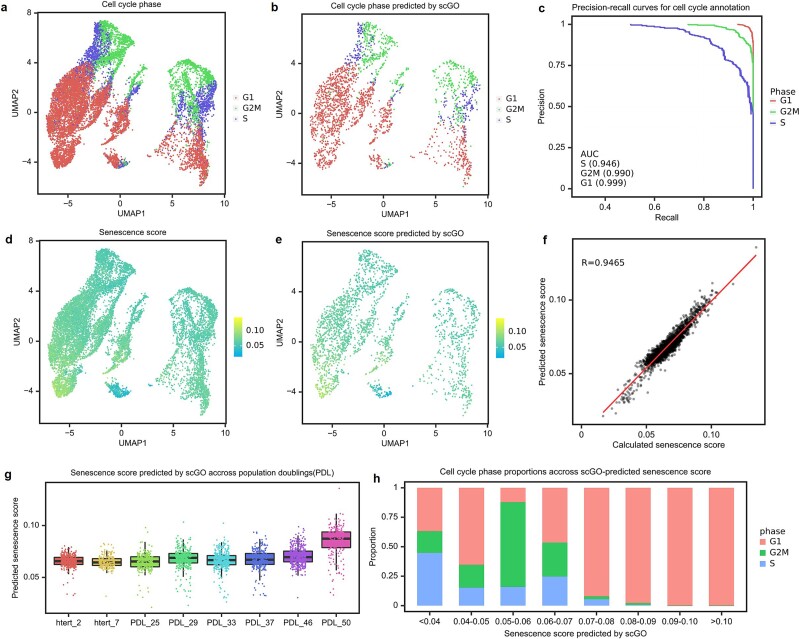
Quantification of cellular senescence. (a, b) Cell cycle stage calculated by cell cycle score (a) and predicted by scGO (b). (c) ROC curve of scGO cell cycle prediction model. (d, e) Senescence score calculated by scoring function (d) and predicted by scGO (e). (f) The scatter plot demonstrates the correlation between the calculated senescence score and the predicted senescence score. Each data point on the plot signifies an individual cell. (g) The distribution of predicted senescence scores in relation to PDLs displayed a progressive rise, indicating an incremental increase across subsequent generations. (h) The statistics of cell cycle stages across the range of senescence scores.

Establishing reliable methods to identify senescent cells remains a bottleneck in understanding the roles of senescence in physiology and disease [83]. In this context, we focused on discerning cell cycle stages and quantitatively assessing cellular senescence utilizing the scGO model. Our approach employed scRNA-seq data derived from WI-38 human lung fibroblasts undergoing replicative senescence [84]. The wild-type and telomerase reverse transcriptase–immortalized WI-38 cells were consistently cultured with periodic sampling for scRNA-seq to capture the replicative senescence process. scGO archived an ROC-AUC of 0.94 for identifying the S stage, 0.999 for the G1 stage, and 0.99 for the G2M stage (Fig. 7a–c).

We then adapted the scGO model into a regression framework to quantify cellular senescence. Cellular senescence scores (Fig. 7d) were calculated using Seurat, following established methodologies [84, 85]. The scGO regression model, trained on the designated dataset, accurately predicted cellular senescence scores in the test set (Fig. 7e), showcasing a robust Pearson’s *R* correlation coefficient of 0.9456. (Fig. 7f). Predicted senescence scores increased progressively with population doublings (PDLs) (Fig. 7g). Upon examining the cell cycle stages in relation to the predicted senescence scores, it was observed that cells with higher senescence scores gradually arrested at the G1 stage (Fig. 6h), consistent with prior studies [86–88].

Next, we applied *in silico* gene perturbation analysis to the scGO regression model to identify senescence-associated genes. In total, scGO identified 399 senescence-associated genes (Supplementary Fig. 10). Among these identified genes, *PTMA*, *RPS20*, *ACTG1*, and *AP5MC3*, displayed a notable decline in their expression levels as senescence progressed (Supplementary Fig. 10a). Conversely, another subset of genes, including *CD44*, *TIMP2*, *TRAM1*, and *DKK3*, exhibited a noticeable increase in their expression levels during the senescence progression (Supplementary Fig. 11).

## Discussion

Accurate and interpretable annotation of cell types is essential for advancing our understanding of cellular functions and heterogeneity in biological systems. However, conventional deep learning approaches often lack the transparency needed for meaningful biological interpretation. In this study, we introduced scGO, a biologically informed deep learning method designed for precise and interpretable cell status annotation. Within the scGO framework, each node represents essential biological entities, including genes, TFs, GO terms, and cell types (Fig. 1a). This unique feature grants scGO inherent transparency, allowing for interpretable predictions. By tracing back the weights and gradients of the scGO model, we can assess the individual contributions of genes or GO terms to the prediction results, thus enriching our comprehension of the biological significance underlying the model’s findings.

We attribute the high accuracy of scGO to its sparse connectivity and feature prioritization. The sparsity of connections within the scGO model leads to significantly fewer parameters. This reduction offers multiple advantages. Firstly, the simplicity engendered by this sparsity lowers the computational demands, making it more computationally efficient. Secondly, using sparse connections safeguards against overfitting, ensuring that scGO’s predictions remain generalizable and reliable even in complex and noisy biological datasets. Lastly, the lightweight design of scGO ensures ease of retraining and facilitates its application in annotating unlabeled cells during the demultiplexing of scRNA-seq data.

Despite the above advantages, scGO’s reliance on high-quality GO and TF-binding data may present challenges, particularly for species with less well-characterized or incomplete annotations of GO terms and TF-binding profiles. The possibility of missing specific TF–gene connections in particular cells may diminish the interpretability of cell type annotation results. This situation could be addressed with the accumulation of more precise knowledge from future experiments. The sparse connectivity makes scGO a lightweight model. While sparse connectivity enhances computational efficiency, it may limit the model’s capacity to capture complex biological relationships, especially in large and diverse datasets.

Our future efforts will integrate multi-omics data into the scGO framework, incorporating DNA methylation and chromatin accessibility information to provide a more comprehensive understanding of the epigenetic regulation of cell type–specific gene expression. By combining epigenetic regulation patterns with GO and TF-binding data, we aim to enhance the model’s ability to predict cell states and identify potential epigenetic markers for disease diagnosis and therapeutic targets. Ultimately, these advancements will further establish scGO as a versatile and powerful tool for studying cellular heterogeneity and advancing biological and medical research.

## Conclusions

This work establishes scGO as a sparse and interpretable deep learning model for cell status annotation and disease diagnosis. By leveraging GO and TF-binding knowledge, scGO provides transparency behind the annotation results. In addition to cell type annotation, we demonstrate that scGO could be applied to various tasks such as cell disease diagnosis, developmental stage annotation, and cell senescence quantification. Application across mammals and plants showcases its versatile usage in studying cellular heterogeneity.

**Key Points**

- The study introduces scGO, a sparse and interpretable model that integrates Gene Ontology (GO) and transcription factor (TF)–binding potential, demonstrating reduced computational cost compared to fully dense models.
- scGO outperforms state-of-the-art methods in accuracy across diverse datasets and provides interpretable predictions, highlighting key genes, GO terms, and TF-binding events that are critical to its decisions.
- scGO is applied to various biological tasks and species, such as disease diagnosis, therapeutic target discovery, developmental stage prediction, and cell senescence evaluation, showcasing its versatility and practical utility. scGO offers powerful capabilities for *in silico* gene manipulations, allowing for identifying therapeutic targets and discovering functional gene modules.
- scGO is freely available at https://github.com/yulab2021/scGO.

## Supplementary Material

scGO_manuscript_additional_information_final_BiB_12_16_bbaf018

Supplementary_Table1_bbaf018

## Data Availability

All datasets used in this study are listed in Supplementary Table 1. The source code of scGO is available for research purposes at Github: https://github.com/yulab2021/scGO. Online documentation is available at https://yulab2021.github.io/scGO_document.
