# Supplementary material for: scGO: interpretable deep neural network for cell status annotation and disease diagnosis
Source: Brief Bioinform. 2025 Jan 16;26(1):bbaf018. doi: 10.1093/bib/bbaf018 (PMC11737892; doi:10.1093/bib/bbaf018)
Supplement: scGO_manuscript_additional_information_final_BiB_12_16_bbaf018 [file scgo_manuscript_additional_information_final_bib_12_16_bbaf018.docx]

**scGO: Gene Ontology-inspired Deep Neural Network** **for Interpretable Cell Status Annotation**

You Wu^1,5^, Pengfei Xu^1,5^, Liyuan Wang^2,5^, Shuai Liu^1^, Yingnan Hou^2^, Hui Lu^1^, Peng Hu^3,*^, Xiaofei Li^1,4,*^, Xiang Yu^1,*^

^1^Joint International Research Laboratory of Metabolic & Developmental Sciences, School of Life Sciences and Biotechnology, Shanghai Jiao Tong University, Shanghai 200240, China

^2^School of Agriculture and Biology, Shanghai Jiao Tong University, Shanghai, China

^3^Key Laboratory of Exploration and Utilization of Aquatic Genetic Resources, Ministry of Education,

Shanghai Ocean University, Shanghai, China

^4^Shanghai Pudong New Area People’s Hospital, Shanghai, China

^5^These authors contributed equally: You Wu, Pengfei Xu, Liyuan Wang

*Corresponding author: Xiang Yu, [yuxiang2021@sjtu.edu.cn](mailto:yuxiang2021@sjtu.edu.cn); Xiaofei Li, [xiaofeili@sjtu.edu.cn](mailto:xiaofeili@sjtu.edu.cn);

Peng Hu, phu@shou.edu.cn.

**Supplementary Figure 1 | A simplified example of the scGO model structure. (a)** Illustration of the scGO model structure with a simplified example. The genes and TFs were connected to GO terms according to GO annotation knowledge; GO terms directly connect to each cell type. **(b)** Sparse weights utilized in the scGO model. The weight matrices connecting the gene layer and the GO layer, as well as the weight matrices between the TF layer and the GO layer, exhibit sparsity. The weight matrix between the GO layer and the cell layer is dense.


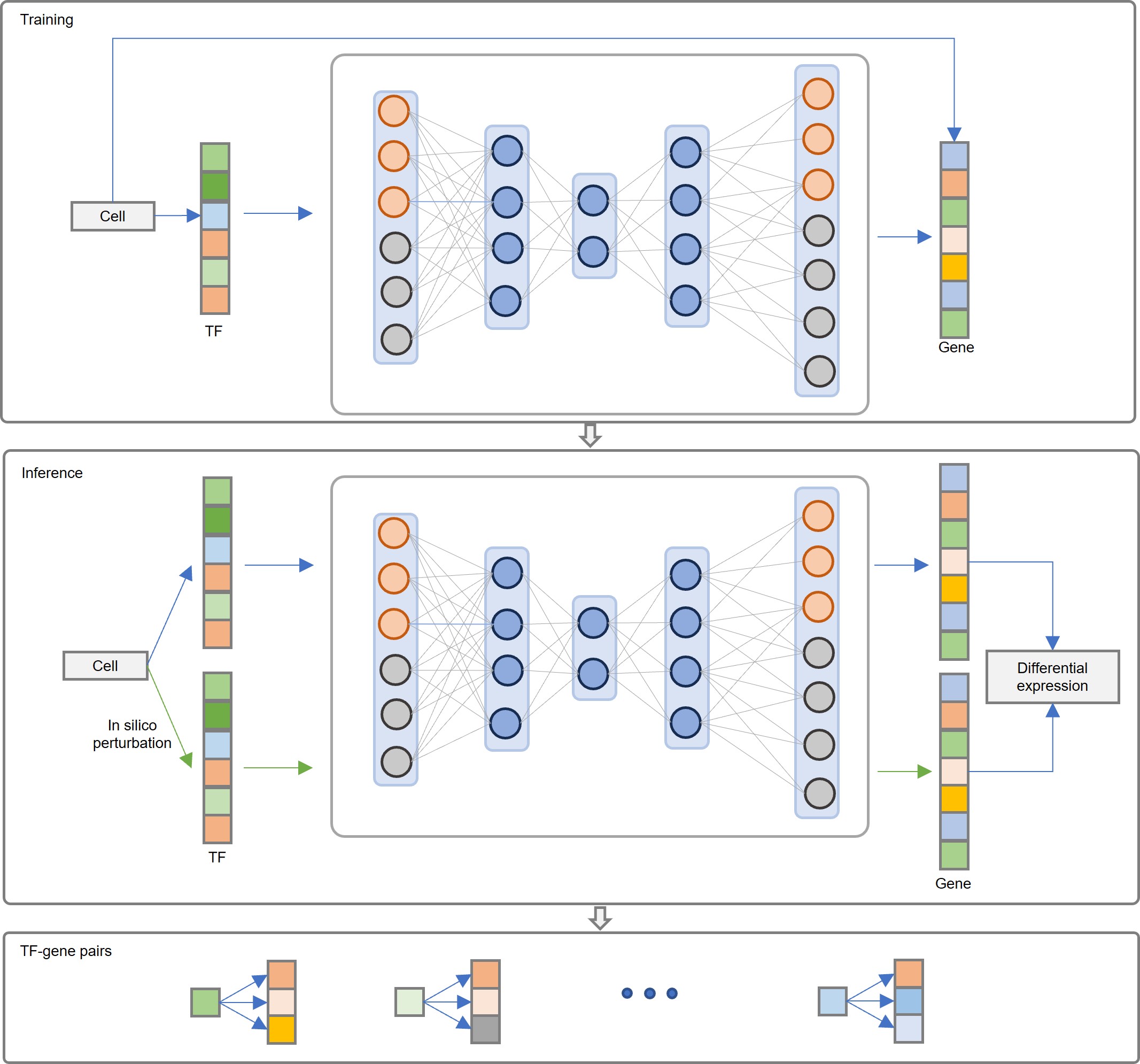


**Supplementary Figure 2 | A schematic overview of de novo TF-target inference mode.** In the training process, the model takes TF and all gene expression as input and output, respectively. Subsequently, *in silico* perturbations are applied to the trained model, and the resulting differential RNA expression is calculated. The genes with the most significant fluctuations are identified as the targets of the perturbed TF.

**Supplementary Figure 3 | Performance and hyperparameters evaluation of scGO. (a)** Variation in memory usage with differing input features. The memory usage slightly increases with larger gene numbers in the input. **(b)** Variation in running time with GO number. The running time is proportional to the number of GO terms used in the scGO model. **(c)** Accuracy of the scGO model under different input features. The scGO model was trained on the Baron dataset. The accuracy reaches its maximum when utilizing 2000 genes as input. As a result, scGO recommends a minimum of 2000 genes for optimal performance. **(d)** Accuracy of the scGO model under different number of GO terms. **(e-f)** Comparison between the training and test accuracies between the dense neural network **(e)** and scGO **(f)**. The dense neural network exhibits a gradual increase in accuracy, culminating in typical overfitting, due to its large parameters. In contrast, scGO swiftly attained high performance and maintained stability without overfitting.


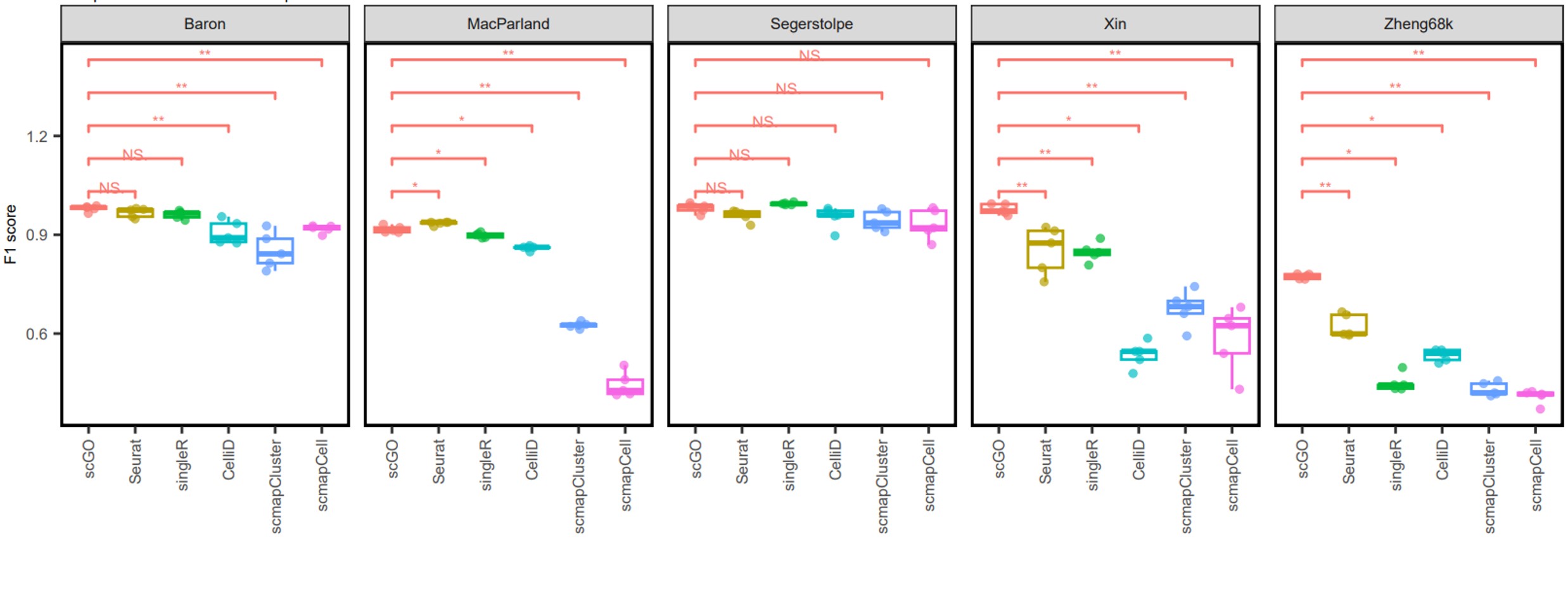


**Supplementary Figure 4 | Performance evaluation of scGO across different datasets** Performance comparison of scGO with Seurat, singleR, CelliD, scmapCluster and scmapCell on five datasets (Zheng68K, Baroon, Segerstolpe, MacParland and Xin). The F1-score is used as the evaluation metric. All statistical analyses use two-sided Wilcoxon tests. The significance levels are as follows: *p <  0.05, **p <  0.01, ***p <  0.001.

**Supplementary Figure 5 | Performance evaluation of scGO across datasets generated from different experimental protocols. (a)** Umap visualization of the panc8 dataset. The data was integrated by Seurat and colored by experimental protocols. **(b)** Umap visualization of the panc8 dataset. The data was integrated by Seurat and colored by cell type. **(c)** Cell type predictions generated by the scGO model, which was trained on a combination of datasets including celseq2, fluidigmc1, smart-seq2, and indrop, and subsequently tested on the celseq dataset. **(d)** Cell type predictions generated by the scGO model, which was trained on a combination of datasets including celseq, celseq2, smartseq2, and indrop, and subsequently tested on the fluidigmc1 dataset. **(e)** Cell type predictions generated by the scGO model, which was trained on a combination of datasets including celseq, celseq2, fluidigmc1, and indrop, and subsequently tested on the smartseq2 dataset. **(f)** Cell type predictions generated by the scGO model, which was trained on a combination of datasets including celseq, celseq2, fluidigmc1, and smartseq2, and subsequently tested on the indrop dataset.

**Supplementary Figure 6 | Probability distribution predicted by scGO for both known and novel cell types. (a)** The representation of the probability distribution for known cell types is structured as a matrix, where each row denotes an input cell type and the columns delineate the predicted probabilities for various cell types. High probabilities align with the diagonal, signifying accurate predictions of the true cell types. In contrast, probabilities in other positions across the matrix are comparatively lower. This distinct pattern demonstrates the scGO model's precision in accurately predicting each cell type with a high level of confidence. **(b)** The prediction probability distribution for novel cell type (the plasma cell). In scenarios where scGO encounters a novel cell type it hasn’t previously encountered, scGO consistently assigns low probabilities across the various known cell types.

**Supplementary Figure 7 | *In silico* gene perturbation analysis and therapeutic targets discovery.** **(a)** *In silico* gene perturbation. The process involves activating or inhibiting individual genes within normal cells to assess their influence on cell type transitions towards a diseased state. **(b)** *In silico* treatment analysis. This process entails activating or inhibiting individual genes within diseased cells to assess whether such interventions can prompt a shift of the diseased cells towards a normal status.

**Supplementary Figure 8 | Umap visualization of cell embeddings from each layer of scGO. (a)** Cell embeddings from the input gene layer. **(b)** Cell embeddings from the TF layer of scGO. **(c)** Cell embeddings from the GO layer. As the cells progress through each layer, the model systematically separates them to derive distinct embeddings that effectively distinguish between different cell types.

**Supplementary Figure 9 | Heart diseases associated genes identified by scGO. (a)** The genes identified by scGO whose *in silico* activation would shift normal cells towards a hypertrophic state. **(b)** The genes identified by scGO whose *in silico* deletion would shift normal cells towards a hypertrophic state. **(c)** The genes identified by scGO whose *in silico* activation would shift normal cells towards a dilated state. (**d)** The genes identified by scGO whose *in silico* deletion would shift normal cells towards a dilated state. TFs were colored in green and genes were colored in light blue.

**
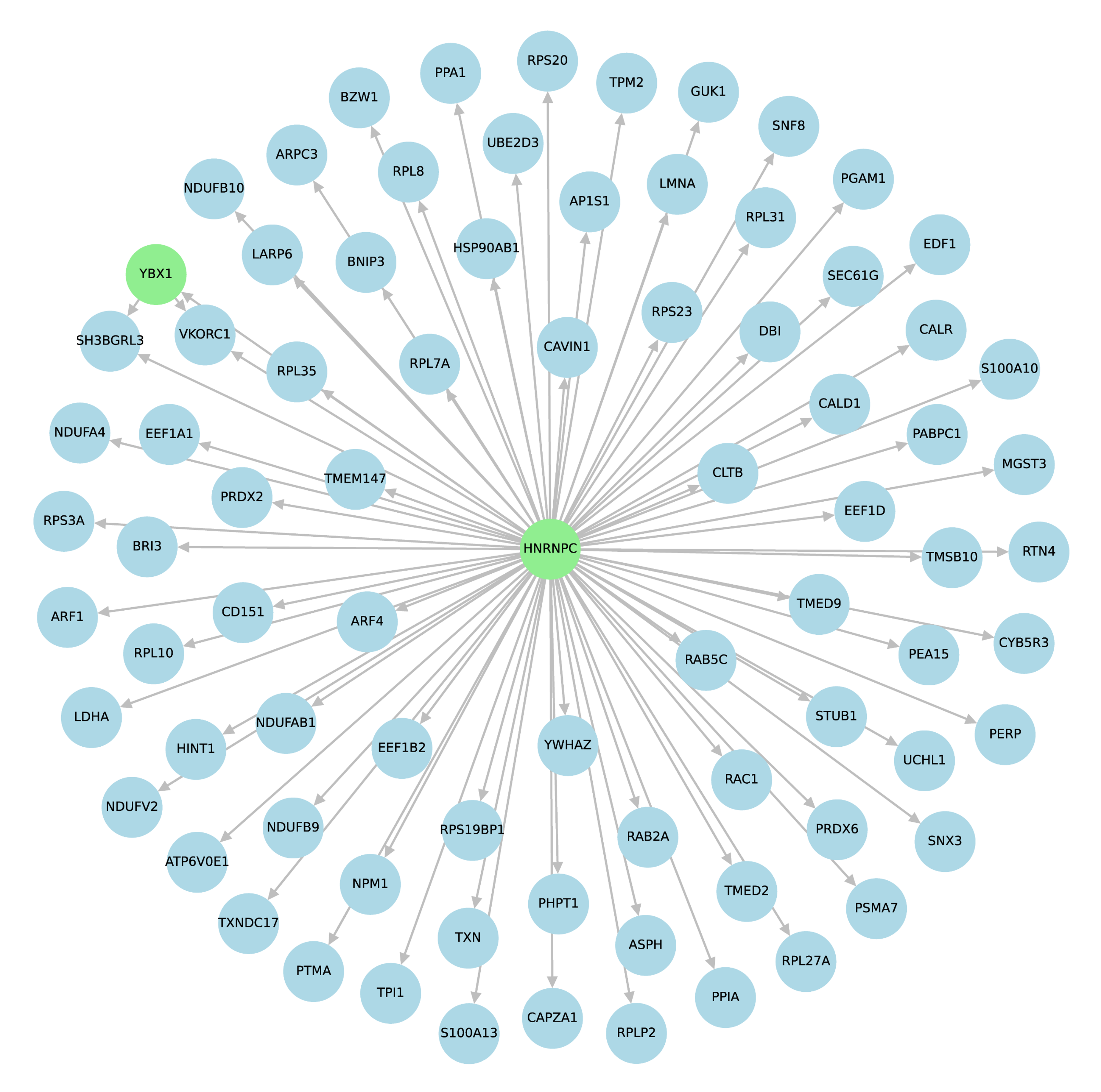
**

**Supplementary Figure 10 | Senescence associated genes identified by scGO.** The graph displayed 81 linked nodes, which is a subset of the identified genes. TFs were colored in green and genes were colored in light blue.

**Supplementary Figure 11 |** **Normalized expressions for senescence-associated genes. (a)** The gene expressions of *PTMA*, *RPS20*, *ACTG1*, and *AP5MC3* showed a decrease during the progression of senescence. **(b)** The gene expressions of *CD44*, *TIMP2*, *TRAM1*, and *DKK3* exhibited a gradual increase throughout the senescence progression. The normalized gene expressions were calculated by scTransform in the Seurat package.
